# Modeling of vertical transmission and pathogenesis of cytomegalovirus in pregnancy: Opportunities and challenges

**DOI:** 10.3389/fviro.2023.1106634

**Published:** 2023-02-03

**Authors:** Gregory W. Kirschen, Irina Burd

**Affiliations:** 1 Department of Gynecology and Obstetrics, The Johns Hopkins Hospital, Baltimore, MD, United States,; 2 Department of Obstetrics, Gynecology and Reproductive Sciences, University of Maryland, Baltimore, MD, United States

**Keywords:** congenital CMV infection, natural killer cell, T lymphocyte, Hofbauer cell, macrophage, placental immunology

## Abstract

In addition to facilitating nutrient, oxygen, and waste transfer between developing fetus and mother, the placenta provides important immune barrier function against infection. Elucidation of the complexity of placental barrier function at the maternal-fetal interface has been greatly aided through experimental model organism systems. In this review, we focus on models of vertical transmission of cytomegalovirus (CMV), a ubiquitous double-stranded DNA viruses whose vertical transmission during pregnancy can lead to devastating neurological and obstetric sequelae. We review the current evidence related to guinea pig and murine models of congenital CMV infection, discuss the possible translatability of a non-human primate model, and conclude with recently developed technology using human placental organoids.

## Introduction

Among its many roles, the placenta serves to protect the developing fetus from potential teratogens and microbes. Congenital infections can occur when the placental barrier is breached, allowing pathogens to enter fetal circulation and exert deleterious effects on growth, organ development, and even survival. Study of congenitally acquired infections has focused on the maternal-fetal interface, where pathogens derived from multiple sources including the bloodstream and reproductive tract cause disruption of the placental barrier and transmission across the fetal membranes, reaching the developing fetus (1).

In this mini-review, we focus on vertical transmission of cytomegalovirus (CMV), a double-stranded DNA herpesvirus that is the most prevalent congenital infection, occurring in an estimated 0.6 to 6% of pregnancies depending on the population (2, 3). The virus can cause a spectrum of teratogenic effects, from asymptomatic infection to neurocognitive impairment, microcephaly, ventriculomegaly, and growth restriction, and is the most common nongenetic cause of sensorineural hearing loss in higher income nations (4, 5). Studies of basic placental biology have been used to decipher how and under what conditions CMV crosses from maternal to fetal circulation and how such effects can occur.

The human placenta forms from cytotrophoblasts creating anchoring villi into the uterine decidua basalis beginning during the second and third weeks of pregnancy (6). Humans have hemochorial, villous, discoid placentas, in which fetal and maternal circulations are separated by a single layer of trophoblast cells, facilitating efficient exchange of oxygen and nutrients (7). Indeed, the placenta becomes the sole source of gas, nutrients, antibodies, and waste exchange between maternal and fetal circulation (8). This also renders the fetus vulnerable to potentially harmful substances and pathogens that are capable of placental crossing, especially through such a thin maternal-fetal interface.

## Current understanding of cytomegalovirus vertical transmission

**FIGURE 1:**
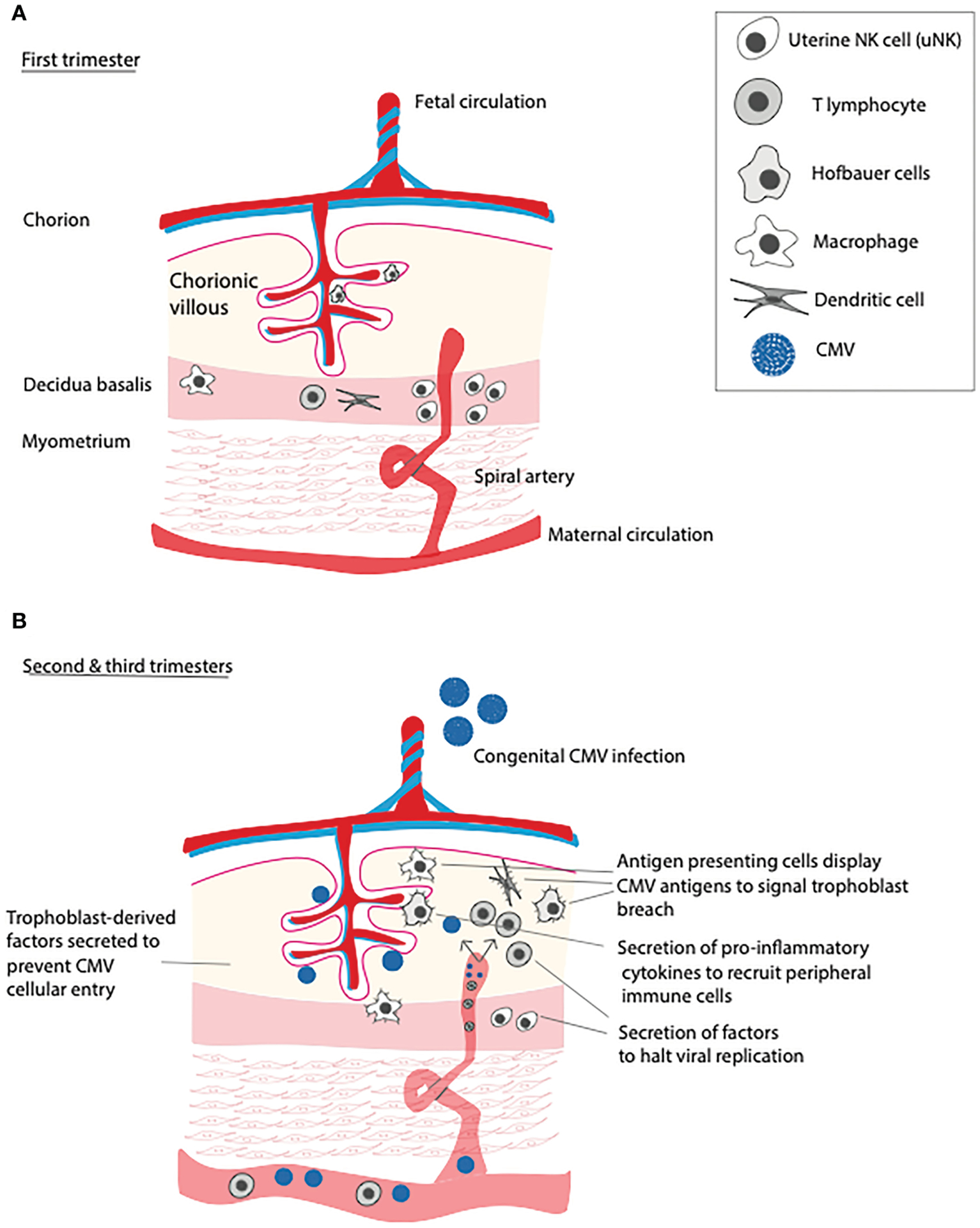
Immune function of the placenta over gestational time and in response to primary CMV infection. (A) In the first trimester, uterine NK cells (uNK) remodel spiral arteries and defend against viral infections that threaten to invade into the chorionic villi. (B) In the second and third trimesters, numbers of uNK cells dwindle, leading increased placental susceptibility to primary CMV infection. Upon infection, placental T cells and antigen presenting cells (APCs) incudling dendritic cells, macrophages, and Hofbauer cells, recruit peripheral T lymphocytes, inducing placental inflammation.

To understand how CMV breaches the placental barrier, it is first important to understand the basics of placental immunology. Maternal leukocytes lie within the decidua, and within the chorionic villi, fetally-derived macrophages known as Hofbauer cells are present (9). In the first trimester of pregnancy, about one third of decidual cells are leukocytes, which can be subdivided into uterine natural killer (uNK) cells, macrophages, dendritic cells, and T lymphocytes (both natural killer and regulatory) (9, 10). In humans, uNK cells represent the most abundant placental leukocyte in early pregnancy, diminishing in number by term, and under physiological conditions are responsible for spiral artery remodeling *via* disruption of vascular smooth muscle cells and extracellular matrix, critical steps for proper placentation (11, 12). In humans, CMV replicates in the decidua, and so it is unsurprising that the first line of defense against CMV are decidual CD8+ effector memory T cells and natural killer (NK) cells, with apolipoprotein B mRNA-editing enzyme catalytic polypeptide 3A (APOBEC3A) serving to curb viral replication (1). In the setting of maternal viral infection, uNK cells are largely responsible for limiting spread into fetal circulation, likely explaining the low rate of vertical transmission of CMV in the first trimester (Figure 1) (13). Indeed, uNK cells have been shown in co-culture experiments to efficiently eliminate human CMV-infected autologous fibroblasts using immune synapses (13). On the other hand, Hofbauer cells (CD68+, CD64+, CD32+, CD16+), upon encountering viral pathogens, become polarized and secrete pro-inflammatory cytokines to recruit peripheral immune cells to the site of infection, also inflicting damage to the placenta in the process and likely leading to a host-inflicted compromise of the placental barrier (14).

As alluded to above, early pregnancy is a particularly immune-privileged time period during gestation, due in large part to the maternal innate immune system. In fact, following primary infection with CMV, vertical transmission of the virus occurs in 30–40% of cases during the first and second trimesters, with up to 70% of cases being transmitted in the third trimester (15, 16). The reasons why advancing pregnancy is associated with increased susceptibility to vertical transmission of CMV are likely multifactorial. Decreasing numbers of uNK cells in addition to CMV evasion of NK cell recognition *via* downregulation of MHC class 1 complexes and upregulation of MHC decoy UL18 on infected NK cells renders the placenta more vulnerable to pathogenic invasion (17).

## Experimental animal models of congenital CMV vertical transmission

Our understanding of the mechanisms by which viruses evades the cell-mediated immune response resulting in vertical transmission and human fetal infection has been greatly aided through the development of model systems. In terms of model organisms, three major animal models have emerged in this field of investigation: guinea pig, mouse, and non-human primate. The guinea pig model of congenital CMV infection came about after the findings by Connor and Johnson reported in 1976 that direct guinea pig CMV (gpCMV) inoculation in weanling guinea pigs resulted in high-titer infection in the salivary glands, thymus, and resulted in multifocal inclusion cell encephalitis with intracerebral inoculation (18). Like humans, guinea pigs also exhibit hemochorial placentation, although they utilize a labyrinth rather than villous strategy, with a dense meshwork of maternal and vascular channels (7, 19). Transplacental transmission of gpCMV in guinea pigs was subsequently demonstrated two years later, with successful fetal infection occurring in 24% of pregnancies in which maternal primary infection occurred during what would correspond to the second and third trimesters of human gestation (20). Since these foundational studies, investigators have used the guinea pig model to demonstrate that gpCMV enters host cells *via* a glycoprotein complex whose receptor is PDGFRA (21, 22).

The gpCMV pentameric complex (PC) consisting of virion envelope proteins gH, gL, UL128, UL130, and UL131A has been the focal point in the development of a vaccine against human CMV (hCMV). Specifically, Schleiss et al. used the the guniea pig model of congenital CMV infection to show that a two-dose vaccine series with live, attenuated gpCMV (either genetically modified to lack PC or with intact PC) protected against maternal viremia compared to placebo (23). Importantly, the vaccine harboring the PC led to improved immunogenicity and decreased vertical transmission in the model (23). Another vaccination strategy has been to use purified gpCMV glycoproteins to provoke a host antibody response prior to conception (24). Despite these advances, we still lack a vaccine against CMV that is approved for use in humans to protect against congenital CMV infection.

Understanding the pathogenesis of congenital CMV vertical transmission has been further aided by mouse models, which may improve our ability to engineer targeted and effective vaccination or treatment of the disease in humans. Like humans and guinea pigs, mice also display hemochorial placentation and like guinea pigs and other rodents, mice use a labyrinth rather than villi (7). Vertical transmission of murine CMV (mCMV) was first demonstrated in 1979, just as other animal models of the disease were coming into the arena. Chantler et al. inoculated young adult non-pregnant mice with high-titer mCMV intraperitoneally, then waited one year and allowed them to become pregnant before studying their embryos (25). They found that cultured embryonic cells contained mCMV particles and mCMV-associated histopathological changes.

Unlike the guinea pig model, however, the murine CMV model has been limited back resistance to vertical transmission in immunocompetent mice. For this reason, an immunocompromised (severe combined immunodeficient, SCID) mouse model has been the staple for congenital CMV investigation in mice. For instance, Woolf et al. (26) performed time pregnancy in SCID mice inoculated with high-titer mCMV on embryonic day 0 (E0) or E3-E5 and demonstrated both transplacental cross of mCMV and characteristic fetal changes including microcephaly and growth restriction along with upregulated fetal cerebral interleukin-1alpha (IL-1alpha) expression suggestive of neuroinflammation. Despite its benefits at aiding to elucidate the pathogenesis of congenital CMV disease, the SCID mouse model of vertical transmission of CMV is limited by the immunocompromised nature of the host. In the immune-intact condition, the maternal immune response to viral pathogens relies importantly on both peripheral and decidual CD8+ effector-memory T cells, which likely serve overlapping and complementary functions of killing virus-infected cells and effecting a memory response, respectively (27, 28). Thus, the SCID mouse model of congenital CMV disease may serve as a good representation of infection under immunocompromised conditions (for instance, in pregnant women co-infected with HIV/AIDS or who are transplant recipients on immunosuppression), but likely does not recapitulate the complex interplay between host and pathogen in immunocompetent individuals.

Approaching the human condition more closely would likely require a non-human primate (NHP) model of congenital CMV infection. Similarities between humans and NHPs with regard to pregnancy anatomy and physiology include both relying on hemochorial, villous discoid placentation, and having similar immune systems (7). While no NHP model of *congenital* CMV has yet been described, we do have a basic understanding of CMV pathogenesis in NHP in general, based on several seminal studies.

Rhesus CMV (RhCMV) infects rhesus mecaques (*Macaca mulatta*), and thus this host-pathogen interaction has been utilized experimentally to model the disease in humans. Lockridge et al. (29) exposed heatlhy juvenile rhesus mecaques to oral or intravenous RhCMV and observed clinical symptoms, plasma levels, and immune response following inoculation. They found that inoculated animals exhibited splenic tissue that was polymerase chain reaction (PCR) positive for CMV DNA in all animals examined, and multiple other tissues (pancreas, ileum, kidney, lung, thymus, submandibular gland and mesenteric lymph nodes in one animal. In addition, a monocytosis was noted by 4 weeks post injection (wpi) accompanied by a decreased CD4/CD8 T lymphocyte ratio at 2 wpi. Further, anti-RhCMV IgG antibodies were first detected at 2 wpi, with a majority of animals exhibiting a response by 4 wpi and a peak of IgG titers between 10 and 25 wpi. Clinically, IgG avidity testing is used to temporally differentiate acute primary infection from distant primary infection. Low CMV IgG avidity indicates primary infection within the past 3 to 4 months, while high avidity excludes recent infection within the prior 3 months (30). In rhesus macaques, increased avidity was noted between 2 wpi and 25 wpi, consistent with findings in humans (29). Investigating the viral replication of RhCMV in rhesus epithelial cells, Lilja and Shenk (31) identified genetic loci implicated in viral entry and efficient viral replication. These studies aid in our understanding of CMV infection and pathogenesis in an immunocompetent postnatal host closely related to humans.

While we lack a robust evidence base for congenital CMV transmission in NHP models, there are lessons to be learned from vertical transmission primate research related to other infectious diseases. For instance, Terzian et al. identified natural Zika virus (ZIKV) infection among two species of NHP, *Callithrix* and *Sapajus* species from Brazil (32). ZIKV infection induces both humoral and cell-mediated immune responses in NHP, and transplacental transmission of this virus in pregnant NHPs leads to fetal cerebral white matter lesions and periventricular gliosis as well as axonal and ependymal damage as measured histopathologically on autopsy (33, 34). This fundamental work in congenital ZIKV infection has led to a preclinical study in rhesus macaques testing sofosbuvir, a viral RNA synthesis inhibitor (currently Food and Drug Administration, FDA, approved for the treatment of hepatitis C), in an effort to prevent vertical transmission. Gardinali et al. (35) inoculated healthy, pregnant macaques with a viral suspension of ZIKV strain ES 2916/2015/ID 250 during the first and second trimesters of gestation. They then treated half the animals with sofosbuvir (or no treatment as control) and measured maternal weight, creatine kinase (CK) elevation, rash, and fetal death. They observed similar clinical courses, including weight loss, CK elevation, rash, and fetal death in both groups. The untreated group exhibited placental calcifications, with variable fetal manifestations from mild abnormalities to severe congenital ZIKV-associated defects (e.g. skull and thorax abnormalities) and fetal death. Immune phenotyping in untreated dams included high numbers of Hofbauer cells in untreated but not treated dams, and lower numbers of myeloid-derived suppressor cells (MDSCs) in untreated compared to treated dams in placental samples, While the untreated group exhibited ZIKV RNA in placental samples, ZIKV RNA and replication were undetectable in sofosbuvir -treated dams. Importantly, sofosbuvir treatment protected four fetuses against ZIKV transmission, pointing to this pharmacological agent as a potentially viable option for ZIKV congenital infection prophylaxis, Whether similarly focused antiviral interventions would prevent spread of CMV in a primate preclinical model remains to be determined. These studies provide mechanistic insight into the pathogenesis of vertical transmission of viral vectors as well as suggesting how such infections may be prevented, yet more work will be needed to validate these findings in humans and to use these results for the development of targeted therapeutics.

## Placental organoids to model viral infection

**FIGURE 2:**
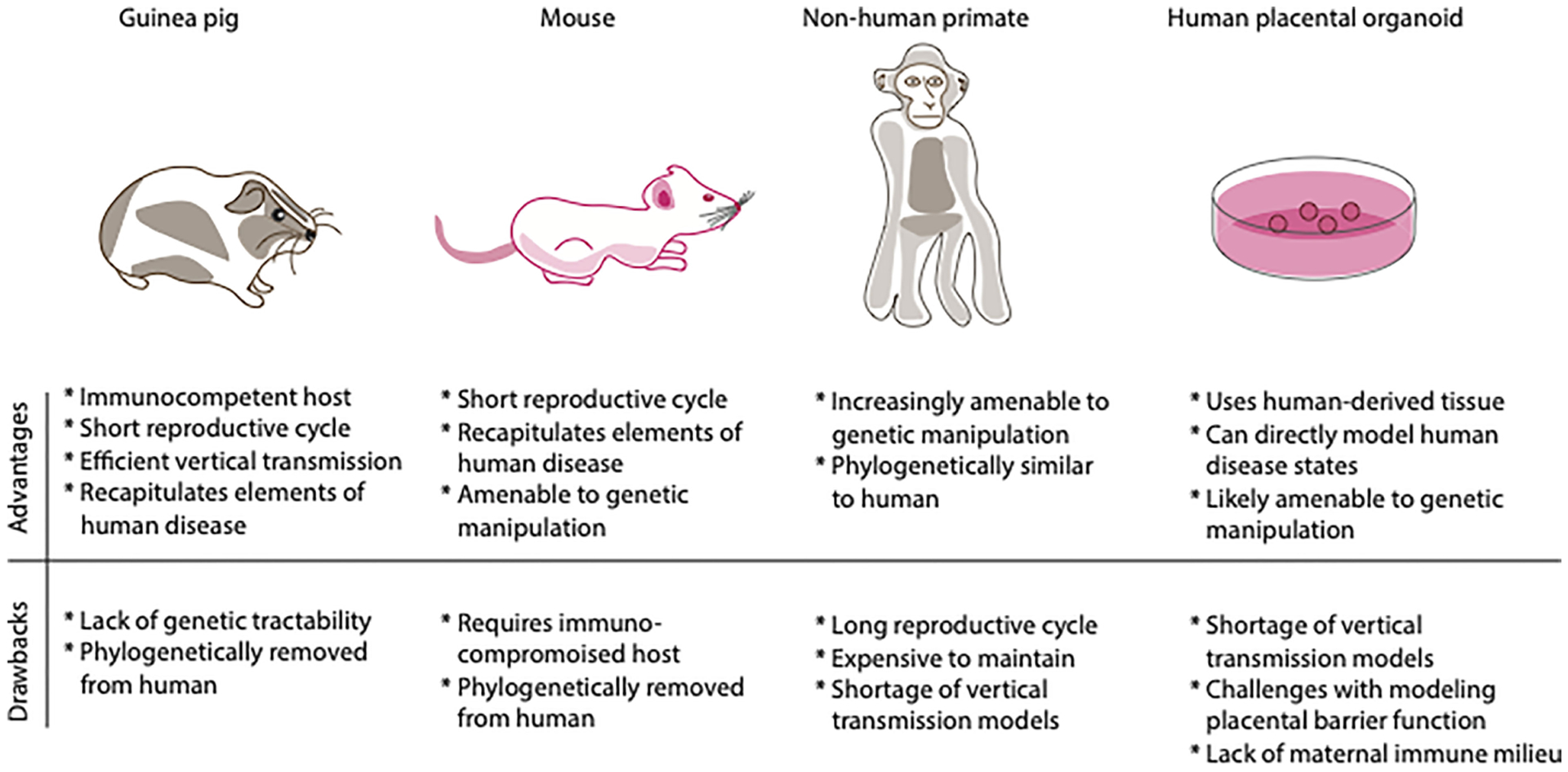
Comparison of relative advantages and drawbacks of various models of vertical transmission. Shown above are depictions of the different model systems of congenital infection. Shown below is a table of the relative advantages and drawbacks of each model system, emphasizing immune system, genetic tractability, life cycle, and cost.

We have reviewed the most widely used model organisms for CMV vertical transmission and pathogenesis, highlighting the relative strengths and limitations of each (see Figure 2 for summary). One criticism of any animal model is that it can never fully replicate the physiology of human tissue. An emerging technology that goes beyond animal modeling and may be used to study vertical transmission of viral pathogens (as well as a myriad of other questions of mammalian pregnancy physiology) is the human placental organoid. Once the native microarchitectural structure of the human placenta was characterized and the technology to isolate and culture trophoblast cells became available, it became possible to design models of human-derived tissue of *in vitro* placentation. The first human trophoblast organoids consisted of a two-dimensional layer of villous cytotrophoblast and extravillous trophoblast cells (36). This *in vitro* system displays similar transcriptomic and DNA methylation profiles as compared to *in vivo* placenta, forms placental villi-like structures, and produces hormones and proteins much like the *in vivo* placenta (36). More recently, the same group of investigators has established a three-dimensional (3-D) human trophoblast-derived organoid (37). Using first trimester derived human placental tissue, these investigators dissected off chorionic villi, digested the tissue and deposited the digested tissue in Matrigel droplet, with organoids forming over the ensuing days (37).

Independently, Karvas et al. (38) developed a trophoblast organoid model of the human placenta using human trophoblast stem cells (hTSCs). They found that hTSCs seeded into 3D Matrigel droplets form 3D organoids with similar tissue architecture and human chorionic gonadotropin (hCG) production as *in vivo* human placenta. These investigators finally exposed their 3D trophoblast organoids to the emerging pathogens ZIKV and SARSCoV-2 to test placental transfer. They found that both viruses were successfully able to enter trophoblast cells and produce key surface proteins, demonstrating tractability for modeling of human placental infection *in vitro*. A subsequent investigation employing human placenta-derived trophoblast organoids and dedicua organoids discovered that both types of organoids secrete cytokines and chemokines, and that trophoblast organoids constitutively secrete antiviral type III interferon IFN-l2 (39) To address the question of how hCMV is transmitted across the placenta and how the placenta responds immunologically to this invader, Yang et al. (39) infected trophoblast and decidua organoids with hCMV strains AD169r (GFP-tagged) and TB40E (mCherry-tagged), and found that while trophoblast organoids, representing the fetal contribution to the placenta, were highly resistant to infection, decidual organoids, representing the maternal contribution, were susceptible to infection, as quantified by high GFP and mCherry expression in organoids. These results imply a robust innate immune response by trophoblast organoids, and indeed the investigators identified differences in transcriptome profiles between trophoblast and decidual organoids, including a greater number of differentially expressed genes in response to hCMV infection in the former compared to the latter. In particular, hCMV induced H3Y1 (H3.Y Histone 1), KHDC1L (KH Domain Containing 1 Like), members of the PRAME family, and TRIM49A and B (Tripartite Motif Containing 43) in trophoblast but not decidual organoids, potentially explaining the relative resistance to infection in the former.

This set of carefully conducted experiments demonstrates how human placental organoids can be used to dissect the individual contributions of fetal and maternal immune components to defense against viral pathogens, as just one example of the power of this tool. Altogether, these findings open up the possibility of in-depth molecular characterization of human placental response to CMV and other viral pathogens as well as potential high throughput drug screens for prevention of viral pathogen vertical transmission.

While human placental organoids are a new technology with many potential applications, the limits of this technology must be contemplated. One potential drawback is that these organoids are derived from first trimester samples, and as mentioned above, hCMV transplacental transmission most commonly occurs in the latter two trimesters of pregnancy. Another potential limitation is lack of the maternal peripheral immune milieu, including circulating cytotoxic and regulatory T lymphocytes important in host response. It is also unclear whether trophoblast organoids retain any of the native uNK cells or fetal Hofbaur cells of the *in vivo* condition, calling into question whether this model could be used to study the host-pathogen interaction from an immunological standpoint. Thirdly, the integrity of the placental barrier at the maternal-fetal interface has yet to be rigorously tested in this model, leaving open the possibility of a “leaky” or disrupted barrier function *in vitro*. Thus, carefully designed experiments, perhaps co-culturing placental organoids with maternal immune cells prior to viral inoculation, will be needed if these organoids are to be used to model vertical transmission of CMV or other microbes.

## Conclusion

In summary, we have reviewed the current progress towards a model of vertical transmission of CMV, which have been used not only to better understand the disease, but also to provide clues for developing a treatment or even a possible vaccine. For instance, placental organoids can provide a platform for high-throughput drug screening to determine candidate compounds that prevent hCMV entry into cells, viral replication, or viral protein production. Further, *in vivo* animal models can be used to test safety and efficacy of candidate compounds for either prevention (e.g. vaccination or cellular entry inhibition) or treatment of acquired congenital CMV. While more work is needed to be done on this front, with no currently Food and Drug Administration (FDA)-approved treatment or prevention strategy for congenital CMV infection, our current tools and refinements in our ability to approach the *in vivo* human condition will likely allow us to reach these goals.
